# How Much Variance Exists Among Published Definitions of Proximal Junctional Kyphosis? A Retrospective Cohort Study of Adult Spinal Deformity

**DOI:** 10.3390/jcm14155469

**Published:** 2025-08-04

**Authors:** Tim T. Bui, Karan Joseph, Alexander T. Yahanda, Samuel Vogl, Miguel Ruiz-Cardozo, Camilo A. Molina

**Affiliations:** 1 Department of Neurosurgery, Washington University School of Medicine in St. Louis, St. Louis, MO 63110, USA; btim@wustl.edu (T.T.B.); jkaran@wustl.edu (K.J.); ayahanda@wustl.edu (A.T.Y.); vogls@wustl.edu (S.V.); miguelr@wustl.edu (M.R.-C.); 2 Department of Orthopedic Surgery, Washington University School of Medicine in St. Louis, St. Louis, MO 63110, USA

**Keywords:** adult spinal deformity, proximal junctional kyphosis, proximal junctional failure, upper instrumented vertebrae, reoperation, spine, spinal deformity

## Abstract

**Background/Objectives**: We sought to characterize the variance and overlap among definitions of Proximal Junctional Kyphosis (PJK) used in the adult spinal deformity (ASD) literature. PJK is defined as excess in PJK angle, a Cobb angle between the upper-instrumented vertebra (UIV) and a supra-adjacent vertebra (SAV), either one (UIV+1) or two (UIV+2) levels rostral of the UIV. No expert consensus exists for threshold angle or which SAV to use. **Methods**: A total of 116 thoracolumbar fusion patients ≥ 65 years old were reviewed. The UIV+1 and UIV+2 angles were measured. Six definitions of PJK from the literature were evaluated. These definitions were selected based on citation frequency, historical relevance, and accessibility through commonly used databases. Pearson’s Chi-squared and pairwise comparisons were performed to evaluate the distinctness and agreement rates among these definitions. **Results**: The six definitions of PJK were as follows: **[PJK20]** PJK angle ≥ 20° with UIV+2 as the (SAV), **[PJK10]** PJK angle ≥ 10° with a >10° change from pre-op with UIV+2 as the SAV, **[PJK2SD]** PJK angle > 2 standard deviations from average with UIV+1 as the SAV, **[PJK10+10]** PJK angle ≥ 10° with a >10° change from pre-op with UIV+1 as the SAV, **[PJK15]** PJK angle > 15° with UIV+1 as the SAV, and **[PJK30]** PJK angle > 30° with UIV+2 as the SAV, or displaced rod fracture, or reoperation within 2 years for junctional failure, pseudoarthrosis, or rod fracture. [PJK10] and **[PJK2SD]** were the most distinct definitions while **[PJK20]**, **[PJK10+10]**, **[PJK15]**, and **[PJK30]** showed no significant pairwise differences. [PJK2SD] was stringent, while definition **[PJK30]** included unique diagnostic information not captured by other definitions. **Conclusions**: The use of **[PJK20]**, **[PJK10+10]**, **[PJK15]**, or **[PJK30]** is recommended for consistency, with **[PJK15]** presenting the best balance. Stringent **[PJK2SD]** may be beneficial for identifying severe PJK, though with low sensitivity. Overall, PJK definitions must be standardized for the consistent reporting of clinical outcomes and research comparability.

## 1. Introduction

Adult spinal deformity (ASD) is a common spinal disorder that is projected to become increasingly prevalent in the future [1]. ASD may be brought about by multiple etiologies, including degenerative disease, iatrogenic causes (e.g., prior spinal surgery), or conditions such as ankylosing spondylitis [2]. Regardless of the cause, ASD often must be addressed via long-segment posterior pedicle screw and rod constructs. These surgeries can be quite effective in restoring proper alignment and promoting adequate fusion. However, extensive rigid constructs also carry the risk of complications, notably proximal junctional kyphosis (PJK), broadly defined as kyphosis that develops at the interface of the cranial end of the construct and the adjacent mobile segments [1,3,4]. The prevalence of PJK after surgery for ASD may lie between 20 and 40%, though some sources report rates as high as 61% [5,6,7,8].

While a global definition of PJK is consistent among publications, various authors have put forth different nuanced criteria [3,5,9,10]. In general, PJK is usually defined as an excess PJK angle, a Cobb angle formed between the upper-instrumented vertebra (UIV) and a supra-adjacent vertebra (SAV) either one (UIV+1) or two (UIV+2) vertebral levels above the UIV. Depending on the study, a Cobb angle of at least 10–20 degrees is required for the diagnosis of PJK [3,5,9,10]. Different cutoff values for PJK angle have been analyzed with respect to their prognostic value as predictors of postoperative pain, functional recovery, or the need for revision surgery [11,12,13]. Nonetheless, the results have not supported one strict definition of clinically significant PJK and no expert consensus currently exists as to the ideal magnitude to use for the PJK angle or which SAV should be used.

To that end, we used a large cohort of patients ≥ 65 years of age who underwent thoracolumbar fusions for ASD to better characterize the variance and overlap among multiple existing definitions of PJK used in the literature and to evaluate which definitions may be the most useful to compare across future studies.

## 2. Materials and Methods

**Figure 1:**
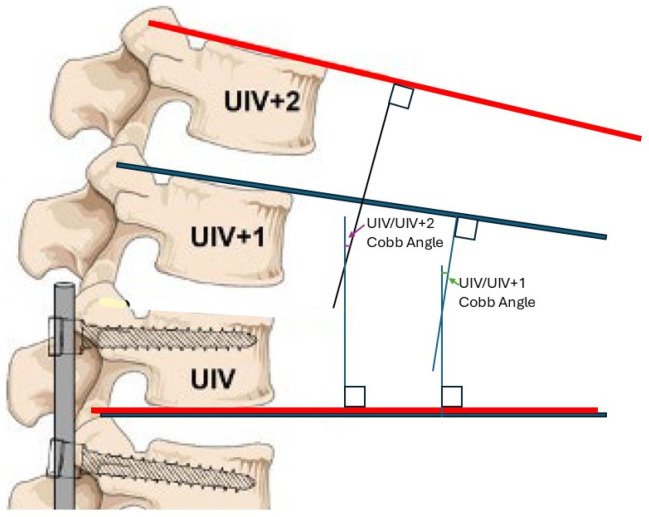
Diagram of the Cobb angle between the upper instrumented vertebrae (UIV) and the UIV+1 (blue) or UIV+2 (red). Figure adapted from Boeckenfoerde et al. with permission per Creative Commons (CC BY) licensing guidelines [14].

Radiographic and demographic data were collected for thoracolumbar fusion from an institutional database for patients ≥ 65 years old with a diagnosis of ASD. Patients who underwent fusion of at least three segments within the thoracolumbar region with pelvic fixation at the authors’ institution between 2014 and 2024 were included, while patients with missing radiographs for the required analysis were excluded. Preoperative and most recent follow-up (≥6 months postoperative) full-body scoliosis radiographs were exported from the patient’s electronic medical record (EMR). The upper instrumented vertebrae (UIV) for each patient were identified from intraoperative/postoperative imaging, and the Cobb angle was measured between the inferior endplate of the UIV and the superior endplate of the vertebrae immediately cranial to the UIV (UIV+1) (Figure 1, blue). A second Cobb angle was measured between the inferior endplate of the UIV and the superior endplate of the vertebrae two levels cranial to the UIV (UIV+2) (Figure 1, red) [14]. Angles were measured from both the preoperative and most recent follow-up radiographs.

A PubMed search with key terms (“proximal junction* kyphosis” AND “definition”) and subsequent exploration of citations yielded 6 criteria for varying definitions of PJK based on different thresholds of the UIV to UIV+1 or UIV+2 Cobb angle [3,5,9,10,15,16]. These definitions were pragmatically selected based on expert opinion, incorporating factors such as citation counts, foundational influence, and prominence in standard database search results. A series of statistical tests were performed with R: A Language and Environment for Statistical Computing version 4.4.1 (CRAN, 2024). A Pearson’s Chi-squared test was performed among the different rates of PJK based on the definitions identified from the PubMed search. A subsequent pairwise comparison of proportions was performed to evaluate the distinctness of each individual definition’s rate in comparison to one another. Rate of agreement among PJK definitions were also analyzed in a pairwise fashion, followed by an evaluation of the proportion of PJK diagnoses that would also be diagnosed by additional criteria. Statistical significance was established by an alpha < 0.05, and 95% confidence intervals (95% CI) were calculated.

## 3. Results

A total of 116 patients met the inclusion criteria, 79 of whom were female (68.1%). The average age of the cohort was 70.9 ± 4.3 years, and the average BMI was 28.9 ± 5.4 (Table 1). Analysis of PJK definitions requiring identification of the UIV+2 decreased the total cohort to 111 patients due to visual obstruction of the UIV+2 vertebrae from either image cutoff or low resolution for 5 patients. For consistency, analysis of all PJK definitions were performed on the 111 eligible patients.

The following six criteria for PJK were identified from the PubMed search: (1) PJK angle ≥ 20° with UIV+2 as the SAV; (2) PJK angle ≥ 10° with a >10° change from preoperative values with UIV+2 as the SAV; (3) PJK angle > 2 standard deviations from average with UIV+1 as the SAV; (4) PJK angle ≥ 10° with a >10° change from preoperative values with UIV+1 as the SAV; (5) PJK angle > 15° with UIV+1 as the SAV; and (6) PJK angle > 30° with UIV+2 as the SAV, displaced rod fracture, or reoperation within 2 years for junctional failure, pseudoarthrosis, or rod fracture (Table 2) [3,5,9,10,16]. These PJK definitions will henceforth be referred to as (1) **PJK20**, (2) **PJK10**, (3) **PJK2SD**, (4) **PJK10+10**, (5) **PJK15**, and (6) **PJK30**. PJK rates, by each definition, were (1) 20.7% (95% CI: 13.8–29.7%), (2) 36.9% (95% CI: 28.1–46.7%), (3) 3.6% (95% CI: 1.2–9.5%), (4) 23.4% (95% CI: 16.1–32.6%), (5) 15.3% (95% CI: 9.4–23.7%), and (6) 10.8% (95% CI: 6.0–18.5%).

Pearson’s Chi-squared testing revealed significant variance among rates of PJK by criteria (*p* = 2.6 × 10^−9^, χ^2^ = 48.7, 95% CI: 0.83–12.8) with a Cramer’s V of 0.27 (95% CI: 0.18–0.34). Post hoc pairwise proportion testing with Holm *p*-value adjustment revealed 5 significantly distinct definition pairs and 10 non-significantly distinct pairs. The distinct pairs were **PJK20**&**PJK2SD** (*p* = 2.63 × 10^−3^, 95% CI: 0.088–0.25), **PJK10**&**PJK2SD** (*p* = 4.86 × 10^−3^, 95% CI: 0.24–0.43), **PJK10**&**PJK15** (*p* = 2.80 × 10^−8^, 95% CI: 0.10–0.33), **PJK10**&**PJK30** (*p* = 1.50 × 10^−4^, 95% CI: 0.15–0.37), and **PJK2SD**&**PJK10+10** (*p* = 4.90 × 10^−4^, 95% CI: 0.11–0.28) (Table 3). In other words, the overall PJK rates for our cohort given by these distinct pairs were significantly different from each other. Meanwhile, the overall PJK rates given by every other pair were not significantly different.

The pairwise agreements of the cohort classification as PJK positive or negative among these six definitions are shown in Table 4. These percentages represented the proportion of patients that were similarly diagnosed as having or not having PJK between the pair of definitions. The pairs with the greatest agreement were **PJK10+10**&**PJK15** at 90.1% (95% CI: 82.6–94.7%), **PJK2SD**&**PJK30** at 89.2% (95% CI: 81.5–94.0%), and **PJK2SD**&**PJK15** at 88.3% (95% CI: 80.5–93.4%) (Table 4). Furthermore, **PJK30**, **PJK2SD**, and **PJK15** had the greatest agreement with reoperation at 82.9% (95% CI: 74.3–89.1%), 79.3% (95% CI: 70.3–86.2%), and 71.2% (95% CI: 61.7–79.2%), respectively.

Among patients with PJK according to a particular definition, the following percentages were identified as having PJK by at least one other definition: (**PJK20**) 91.3%, (**PJK10**) 78.0%, (**PJK2SD**) 100%, (**PJK10+10**) 88.5%, (**PJK15**) 100%, and (**PJK30**) 29.4%. The percentages of each definition’s PJK cohort that met criteria for additional PJK definitions are shown in Table 5.

## 4. Discussion

Discussions of PJK are of much interest in the spinal deformity literature. However, this condition is heterogeneously defined depending on the article and classification criteria used. In this study we used a large cohort of patients who underwent surgical correction of ASD to evaluate six different definitions of PJK identified in the current literature, each broadly cited and readily accessible among the prominent spinal deformity literature. We investigated which definition may encapsulate the most patients in our cohort to determine the definition that may be of most utility when comparing PJK among studies or for potential surgical decision-making. While we found that certain definitions of PJK captured similar patient subsets of our overall patient cohort, there were nevertheless significant differences between multiple criteria as to which patients would qualify as having PJK. In aggregate, our work demonstrates that comparison of results between studies that utilize distinct definitions of PJK may be difficult to interpret and of limited utility.

Only PJK definition **PJK30** directly considered patient outcomes beyond imaging findings by integrating the need for reoperation or instrumentation failure [16]. Hence, we considered it the most clinically relevant definition for a symptomatic PJK. **PJK30**’s close relationship with patient function and greatest agreement with reoperation rates supported its use as a clinical benchmark for comparison with other definitions (Table 4).

**Figure 2:**
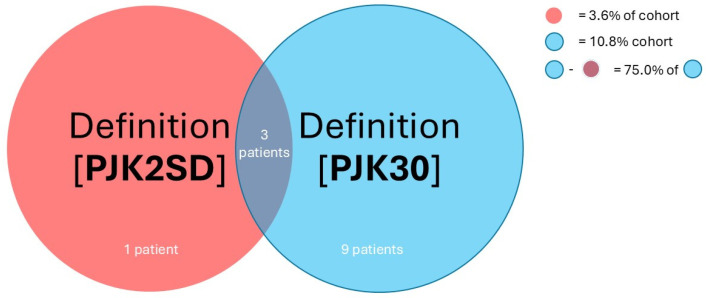
Venn diagram illustration of the overlapping relationship between definition **(3) PJK2SD** and **(6) PJK30**, showing that 75.0% of PJK+ patients by **(6) PJK30** are not accounted for by **(3) PJK2SD**.

Considering the raw rate of PJK-positive patients in the same cohort, definition **PJK2SD** was the strictest for assigning PJK, while definition **PJK10** was the most lenient among the six PJK definitions. The low rate of PJK for definition **PJK2SD** suggests that the dispersion of UIV/UIV+1 angles was functionally small to yield such a stringent diagnosis [9]. Notably, the tightness of distribution of this UIV/UIV+1 angle was relatively more extreme than the already conservative benchmark definition **PJK30**. This means that, in contrast to clinically relevant definition **PJK30** (diagnosing 10.8% of the entire cohort), only 3.6% of the entire cohort experienced the kyphotic angle at the UIV varying outside expected ranges per definition **PJK2SD**. A conservative calculation, assuming all 3.6% of PJK patients per definition **PJK2SD** were also accounted for by definition **PJK30,** found that 66.7% of patients who experienced symptomatic PJK by definition **PJK30** did not meet the threshold for definition **PJK2SD**. Considering that only 89.2% of positive or negative PJK diagnoses from definitions **PJK2SD** and **PJK30** overlapped (Table 4), 75.0% of patients with symptomatic PJK (qualified by definition **PJK30**) were not accounted for by definition **PJK2SD** (Figure 2). It should be noted that definition **PJK2SD** was historically applied to adolescent scoliosis, which features a distinctly different clinical, biomechanical, and pathophysiologic picture than the degenerative ASD featured in our cohort [17].

A comparison of raw PJK rates from definitions **PJK10** and **PJK10+10**, which differ only by usage of the UIV+1 or UIV+2 vertebrae as the cranial vertebrae in Cobb angle measurements, demonstrates that the use of definition **PJK10+10**, and thus the UIV+1 vertebrae, results in stricter criteria for PJK (Table 2) [3,10]. Again, if we were to use definition **PJK30** as the standard for symptomatic PJK, the use of definition **PJK10**, or the UIV+2 vertebrae, could be interpreted as too lenient of a definition in comparison to use of definition **PJK10+10**, or UIV+1.

Closer evaluation of the pairwise overlap between definition **PJK30** and either definitions **PJK10** or **PJK10+10** confirms that definition **PJK10+10**, or the use of the UIV+2, also diagnoses the PJK status of individual patients more similarly to that of definition **PJK30** (Table 4). In fact, definition **PJK10** had the least overlap with definition **PJK30** compared to all other definitions, whereas definition **PJK2SD**, which used extreme deviations of PJK from average values, had the best overlap. This might suggest that, while definition **PJK2SD** is the strictest criteria for PJK, those who met the high threshold to be considered PJK positive by **PJK2SD** were more likely to require reoperation. However, definition **PJK30** also includes a criterion for extreme PJK angle magnitude, which may confound this conclusion.

Chi-square testing with post hoc pairwise proportions analysis and *p*-value adjustment demonstrated that 10/15 (67%) definition pairs were not significantly different when evaluating overall cohort PJK rates (Table 3). Among the five distinct pairs, definitions **PJK10** and **PJK2SD** were most common. Definition **PJK10** was found to be too lenient and definition **PJK2SD** too strict based on raw PJK rates, which could explain why they was often statistically different from the other definitions and limited in practicality. Definitions **PJK20**, **PJK10+10**, **PJK15**, and **PJK30** were not distinct from each other. These results suggest that definitions **PJK10** and **PJK2SD** should be used with caution when consistency across studies is desired, while definitions **PJK20**, **PJK10+10**, **PJK15**, and **PJK30** may be used more interchangeably.

From the evaluation of additional PJK criteria that were encompassed by each of the six definitions, as shown in Table 5, patients diagnosed as having PJK by definition **PJK2SD** would also be sufficiently diagnosed by most other definitions. In the context of definition **PJK2SD**’s stringent nature, this suggests that most other PJK definitions have sufficient coverage and that use of definition **PJK2SD** may be redundant. On the other end of the spectrum, the patients labeled PJK-positive by **PJK30** were often not diagnosed by other PJK criteria. This finding further supports the utility of definition **PJK30,** including capturing unique information that is missed by the remaining PJK definitions.

Overall, our findings suggest that definition **PJK15** most optimally balances the clinical relevance of definition **PJK30** while not yielding statistically different rates of PJK in our cohort. Additionally, definition **PJK15** utilizes the UIV+1 vertebrae, which found greater agreement with definition **PJK30** than when measuring the UIV+2 vertebrae. Definition **PJK15** may also possibly identify PJK earlier than definition **PJK30** because **PJK15**, which relies purely on imaging characteristics, is not contingent on the patient undergoing reoperation, allowing clinicians to identify early PJK that may not have yet progressed to such a degree to necessitate surgical intervention. That said, this hypothesis requires further validation in future studies that incorporate comprehensive clinical outcomes beyond just reoperation rates.

## 5. Limitations and Future Steps

This study has several limitations. First, its retrospective design inherently limits its ability to establish causal relationships between the definitions of PJK and outcomes such as reoperation rates. Secondly, data collection was performed at a single institution, which may reduce the generalizability of our findings to broader populations. Third, the exclusive focus on patients aged 65 and older may not reflect PJK dynamics in younger cohorts undergoing spinal surgeries. An age restriction was implemented for our cohort to focus analysis on elderly adults, who have a greater risk of developing PJK than younger adults [18,19,20,21,22]. Furthermore, younger adults tend to have distinct clinical presentations of ASD from older adults that cannot be fully accounted for by radiographic measurements, so their inclusion could have obscured our findings [2,23]. Another limitation is this study’s reliance on reoperation as a proxy for clinical outcomes. Certainly, other suboptimal clinical outcomes in the setting of PJK such as severe pain, reduced quality of life, decreased activity tolerance, or neurological symptoms could still prove distressing to patients who did not undergo reoperation for PJK. Similarly, patients could have instrumentation failure that received surgery without displaying any of these symptoms. Our reliance on reoperation as the sole surrogate for symptomatic PJK may potentially lead to incongruence between radiographic outcomes and patient-centered outcomes.

Furthermore, some definitions, such as **PJK2SD**, were historically applied to adolescent scoliosis, which presents distinct biomechanical and clinical characteristics compared to adult spinal deformity. These differences may limit the applicability of such definitions to the adult population examined in this study. Further studies should aim to validate these definitions prospectively in multi-institutional cohorts and consider broader clinical outcomes beyond reoperation to provide a more comprehensive understanding of PJK. Finally, it should be noted that no formal non-radiographic analysis was performed for definition **PJK15** to definitively demonstrate its superiority in predicting clinical outcomes compared to other definitions (e.g., Delphi analysis). **PJK15**’s applicability was inferred from its statistical overlap with **PJK30**—a definition with intrinsic clinical outcome considerations—and this concept should be evaluated more thoroughly in future studies through consensus-building efforts as a next step towards definition standardization (e.g., Delphi analysis, Nominal Group Technique, or RAND/UCLA appropriateness method). Exploring whether different PJK definitions might be more appropriate for different patient subgroups (age, fusion length, and etiology) could further refine clinical decision-making.

## 6. Conclusions

This study highlights the variability in PJK definitions and their implications for clinical and radiographic outcomes. Clinically relevant, symptomatic PJK often does not correspond to larger deviations in PJK angle, underscoring the importance of definitions that align with patient outcomes. Among the definitions evaluated, **PJK30** demonstrated itself to be the most clinically relevant with strong alignment to reoperation rates, thus making it a robust option for identifying symptomatic PJK. **PJK15,** while similar to **PJK30**, relies purely on radiographic criteria and thus offers the advantage of enabling the earlier diagnosis of PJK before clinical symptoms become apparent. In contrast, **PJK20** and **PJK10+10**, though statistically viable, are limited by their reliance on UIV +2 measurements which are less practical than definitions that rely on UIV+1 due to the limited visibility or radiographic cutoff that may present when attempting to visualize a more rostral vertebra. These findings support the need for standardized PJK definitions that emphasize both clinical and radiographic relevance to improve consistency in research and surgical decision-making.

## Figures and Tables

**Table 1:** Descriptive statistics of demographics and characteristics of this study’s cohort.

Characteristics	Category	Frequency (%) or Mean (SD)
Sample Size		116
Sex	Female	79 (68.1%)
	Male	37 (31.9%)
Race	White	108 (93.1%)
	Black	7 (6.0%)
	Other	1 (0.9%)
Age (years)	-	70.9 (4.3)
BMI	-	28.9 (5.4)
Smoking History	Never Smoked	68 (58.6%)
	Smoked	48 (41.4%)
Average Follow-Up (days)	-	865.9 (592.9)
Reoperations/Revisions	Yes	27 (28.4%)
	No	89 (83.6%)
Fusion Length	Short (UIV ≤ T10)	49 (42.2%)
	Long (UIV > T10)	67 (57.8%)
Final UIV/UIV+1 angle (°)	-	9.04 (7.04)
Final UIV/UIV+2 angle (°) *	-	13.40 (8.07)
Δ UIV/UIV+1 angle (°)	-	5.15 (9.09)
Δ UIV/UIV+2 angle (°) *	-	7.82 (11.31)

Abbreviations: BMI, body mass index; SD, standard deviation; UIV, upper instrumented vertebrae. * Sampled from 111 viable patients due to 5 having an unclear UIV+2 on imaging.

**Table 2:** Collection of PJK definitions, referenced studies, and rate of PJK within this study’s cohort per definition [3,5,9,10,16].

Number	Abbreviated Name	PJK Definition	Source DOI	Rate of PJK (%)	95% CI
(1)	**PJK20**	Final UIV/UIV+2 ≥ 20°	Bridwell et al., 2013 [3]	20.7	13.8–29.7%
(2)	**PJK10**	Final UIV/UIV+2 ≥ 10° and ΔUIV/UIV+2 > 10°	Glattes et al., 2005 [5]	36.9	28.1–46.7%
(3)	**PJK2SD**	Final UIV/UIV+1 > 2 SD of average Final UIV/UIV+1	Helgeson et al., 2010 [9]	3.6	1.2–9.5%
(4)	**PJK10+10**	Final UIV/UIV+1 ≥ 10° and ΔUIV/UIV+1 > 10°	Lonner et al., 2007 [10]	23.4	16.1–32.6%
(5)	**PJK15**	Final UIV/UIV+1 > 15°	Hyun et al., 2017 [15]	15.3	9.4–23.7%
(6)	**PJK30**	Final UIV/UIV+2 > 30° or displaced rod fracture or reoperation within 2 years for junctional failure, pseudoarthrosis, or rod fracture	Hills et al., 2024 [16]	10.8	6.0–18.5%

Abbreviations: PJK, proximal junctional kyphosis; UIV, upper instrumented vertebrae; SD, standard deviation; CI, confidence interval. UIV/UIV+1 is the Cobb angle between the UIV and the vertebrae 1 level rostral. UIV/UIV+2 is the Cobb angle between the UIV and vertebrae 2 levels rostral.

**Table 3:** Post hoc pairwise comparison of the various definitions with proportional difference (**top**) and Holm-adjusted *p*-values (**bottom**). Statistically significant distinct pairs of definitions are highlighted.

PJK Definitions		PJK Definitions: Pairwise Difference (95% CI) *					
		**PJK20**	**PJK10**	**PJK2SD**	**PJK10+10**	**PJK15**	**PJK30**
	**PJK20**	-	0.16 (0.045–0.28)	**0.17 (0.088–0.25)**	0.027 (−0.082 to 0.14)	0.054 (−0.047 to 0.15)	0.099 (4.1 × 10^−3^–0.19)
	**PJK10**	-	-	**0.33 (0.24–0.43)**	0.14 (0.016–0.25)	**0.22 (0.10–0.33)**	**0.26 (0.15–0.37)**
	**PJK2SD**	-	-	-	**0.20 (0.11–0.28)**	0.12 (0.042–0.19)	0.072 (4.7 × 10^−3^–0.14)
	**PJK10+10**	-	-	-	-	0.081 (−0.022 to 0.18)	0.13 (0.028–0.22)
	**PJK15**	-	-	-	-	-	0.045 (−0.043 to 0.13)
	**PJK30**	-	-	-	-	-	-
PJK Definitions	**PJK Definitions: *p*-Value with Holm Correction**						
		**PJK20**	**PJK10**	**PJK2SD**	**PJK10+10**	**PJK15**	**PJK30**
	**PJK20**	-	0.106	**2.63 × 10^−3^**	1.00	1.00	0.393
	**PJK10**	-	-	**2.80 × 10^−8^**	0.285	**4.9 × 10^−3^**	**1.50 × 10^−4^**
	**PJK2SD**	-	-	-	**4.90 × 10^−4^**	0.0592	0.393
	**PJK10+10**	-	-	-	-	0.697	0.164
	**PJK15**	-	-	-	-	-	1.00
	**PJK30**	-	-	-	-	-	-

Abbreviations: PJK, proximal junctional kyphosis; CI, confidence interval. * 95% CI does not account for Holm Correction in *p*-value Testing.

**Table 4:** Pairwise agreement among PJK definitions representing the percentage (95% CI) of patients diagnosed similarly between a pair of definitions. The three pairs with greatest agreement are highlighted.

PJK Definitions		**PJK Definitions: Percent Agreement (95% CI)**					
		**PJK20**	**PJK10**	**PJK2SD**	**PJK10+10**	**PJK15**	**PJK30**
	**PJK20**	100%	78.4% (69.4–85.4%)	82.9% (74.3–89.1%)	77.5% (68.4–84.6%)	85.6% (77.3–91.3%)	72.1% (62.6–80.0%)
	**PJK10**	-	100%	66.7% (57.0–75.2%)	77.5% (68.4–84.6%)	76.6% (67.4–83.9%)	61.3% (51.5–70.2%)
	**PJK2SD**	-	-	100%	80.2% (71.3–86.9%)	**88.3% (80.5–93.4%)**	**89.2% (81.5–94.0%)**
	**PJK10+10**	-	-	-	100%	**90.1% (82.6–94.7%)**	73.0% (63.6–80.8%)
	**PJK15**	-	-	-	-	100%	79.3% (70.3–86.2%)
	**PJK30**	-	-	-	-	-	100%
	Reoperation	69.3% (59.8–77.6%)	60.4% (50.6–69.4%)	79.3% (70.3–86.2%)	64.9% (55.2–73.5%)	71.2% (61.7–79.2%)	82.9% (74.3–89.1%)

Abbreviations: PJK, proximal junctional kyphosis; CI, confidence interval.

**Table 5:** Percentage of PJK positive patients meeting up to 5 additional PJK criteria.

	Percentage of Additional PJK Definitions Met (%)					
Definition	1	2	3	4	5	Mean (Across Rows)
**PJK20**	91.3	56.5	43.5	17.4	8.70	43.5
**PJK10**	78.0	41.5	26.8	9.76	4.88	32.2
**PJK2SD**	100	100	100	100	50	90
**PJK10+10**	88.5	65.4	42.3	15.4	7.69	43.9
**PJK15**	100	76.5	64.7	23.5	11.8	55.3
**PJK30**	29.4	11.8	11.8	11.8	11.8	15.3

Abbreviations: PJK, proximal junctional kyphosis.

## Data Availability

The data used for this study were obtained from electronic medical records from Barnes-Jewish Hospital (St. Louis, MO) and contain protected health information. Due to institutional policies and patient confidentiality regulations, these data are not publicly available. Data access is restricted to approved researchers in compliance with ethical and regulatory requirements. Please contact Washington University’s Institutional Review Board for further information on data access.

## Abbreviations

The following abbreviations are used in this manuscript:

ASD	Adult Spinal Deformity
PJK	Proximal Junctional Kyphosis
UIV	Upper Instrumented Vertebra
SAV	Supra-adjacent Vertebra
EMR	Electronic Medical Record
BMI	Body Mass Index
SD	Standard Deviation
CI	Confidence Interval
